# Stable Iodine Nutrition During Two Decades of Continuous Universal Salt Iodisation in Sri Lanka

**DOI:** 10.3390/nu12041109

**Published:** 2020-04-16

**Authors:** Renuka Jayatissa, Jonathan Gorstein, Onyebuchi E. Okosieme, John H. Lazarus, Lakdasa D. Premawardhana

**Affiliations:** 1 Department of Nutrition, Medical Research Institute, Danister De Silva Mawatha, Colombo 8, Sri Lanka; 2 University of Washington, Department of Global Health, Seattle, WA 98195, USA; jgorstein@ign.org; 3 Centre for Endocrine and Diabetes Sciences and Thyroid Research Group, C2 Link Corridor, University Hospital of Wales, Heath Park, Cardiff CF14 4XN, UK; Okosiemeoe@cardiff.ac.uk (O.E.O.); Lazarus@cardiff.ac.uk (J.H.L.); PremawadhanaLD@cardiff.ac.uk (L.D.P.)

**Keywords:** iodine schoolchildren, urine iodine, goitre, iodised salt, water iodine, iodine pregnant women

## Abstract

Universal salt iodisation (USI) was introduced in Sri Lanka in 1995. Since then, four national iodine surveys have assessed the iodine nutrition status of the population. We retrospectively reviewed median urine iodine concentration (mUIC) and goitre prevalence in 16,910 schoolchildren (6–12 years) in all nine provinces of Sri Lanka, the mUIC of pregnant women, drinking-water iodine level, and the percentage of households consuming adequately (15 mg/kg) iodised salt (household salt iodine, HHIS). The mUIC of schoolchildren increased from 145.3 µg/L (interquartile range (IQR) = 84.6–240.4) in 2000 to 232.5 µg/L (IQR = 159.3–315.8) in 2016, but stayed within recommended levels. Some regional variability in mUIC was observed (178.8 and 297.3 µg/L in 2016). There was positive association between mUIC in schoolchildren and water iodine concentration. Goitre prevalence to palpation was a significantly reduced from 18.6% to 2.1% (p < 0.05). In pregnant women, median UIC increased in each trimester (102.3 (61.7–147.1); 217.5 (115.6–313.0); 273.1 (228.9–337.6) µg/L (p = 0.000)). We conclude that the introduction and maintenance of a continuous and consistent USI programme has been a success in Sri Lanka. In order to sustain the programme, it is important to retain monitoring of iodine status while tracking salt-consumption patterns to adjust the recommended iodine content of edible salt.

## 1. Introduction

Iodine is a micronutrient that primarily acts through the thyroid gland and its two hormones (thyroxine and triiodothyronine), and it is vital to the integrity of many physiological functions in the human body [1,2]. Iodine deficiency may affect multiple aspects of human development (including intrauterine physical and neurological development), linear growth, and physiological organ function. Organs such as the brain and nervous system are particularly vulnerable in their formative stages during intrauterine life [1,2]. Fortunately, iodine deficiency is relatively easy and inexpensive to prevent through universal iodisation of all edible salt. This is a pure food-chain effect, beginning with soil erosion and leading to environmental iodine deficiency, and a lack of iodine sources in our typical diet. Iodised salt was first introduced in Switzerland in 1922 [2,3] and has been used in many previously iodine-deficient countries with good results [4]. The restoration of iodine sufficiency in many of these countries has been a major public-health triumph facilitated by the United Nations Children’s Fund (UNICEF), World Health Organisation (WHO), and International Council of Control Iodine Deficiency Disorders (ICCIDD, now named Iodine Global Network (IGN)). Statutory regulations enforcing universal salt iodisation (USI) were implemented by regulatory authorities in each country [5]. Sri Lanka is one such country that has successfully adopted a USI programme since 1995.

### History of Iodine Deficiency and Its Management in Sri Lanka

Bennet and Pridham first referred to the existence of endemic goitre along the coast of Galle in the southern province of Sri Lanka in 1849 [6]. However, the link between poor iodine consumption and endemic goitre was first recognised only in the 20th century in a WHO study that confirmed high goitre rates, an iodine-poor diet, and low iodine concentrations in drinking water in 1950 [7]. Mahadeva and his group in 1960 identified a “goitre belt” extending across the western, central, southern, sabaragamuwa, and uva provinces in Sri Lanka [8]. The high annual rainfall in these regions led experts to believe that iodine was “leeched” from the soil, leading to iodine deficiency. At that stage, almost no goitre had been identified in the northern, eastern, and north-western provinces [9]. However, in 1986, Fernando et al. described a high goitre rate of 18.8% in schoolchildren in 17 of 24 districts in Sri Lanka—a variable prevalence of 6.5% in the Matale district and 30.2% in the Kalutara district [10]. This study used palpation as the method of goitre assessment, and was the first to recognise iodine deficiency as a major public-health problem.

USI was introduced nationwide by the government in 1995 by statutory regulation [11]. This legislation banned the sale of non-iodised salt for human consumption, thus ensuring access to iodised salt to all consumers in the country. Potassium iodate was used as the vehicle of iodine supplementation, and added to salt at an optimal concentration of 50 ppm at producer level and 25 ppm at consumer level. The national reference laboratory for monitoring USI was established at the Medical Research Institute (MRI) in 2000 with the aid of UNICEF. This laboratory has the dual role of monitoring USI and of assessing its clinical impact by performing periodic national iodine surveys (NISs). External quality control is linked to the EQUIP programme of the Centers for Disease Control (CDC), Atlanta, Georgia, USA [12].

We review and describe the iodine-nutrition status in Sri Lanka by utilising serial datasets from the four national iodine surveys carried out by the MRI between 2000 and 2016. We assessed the success of USI in Sri Lanka in relation to global indicators of population iodine status, i.e., median urine iodine concentration (mUIC), total goitre prevalence rates (TGRs), and household salt iodine (HHIS) consumption.

## 2. Methods

### 2.1. Available Data Sources for Analysis

**Figure 1:**
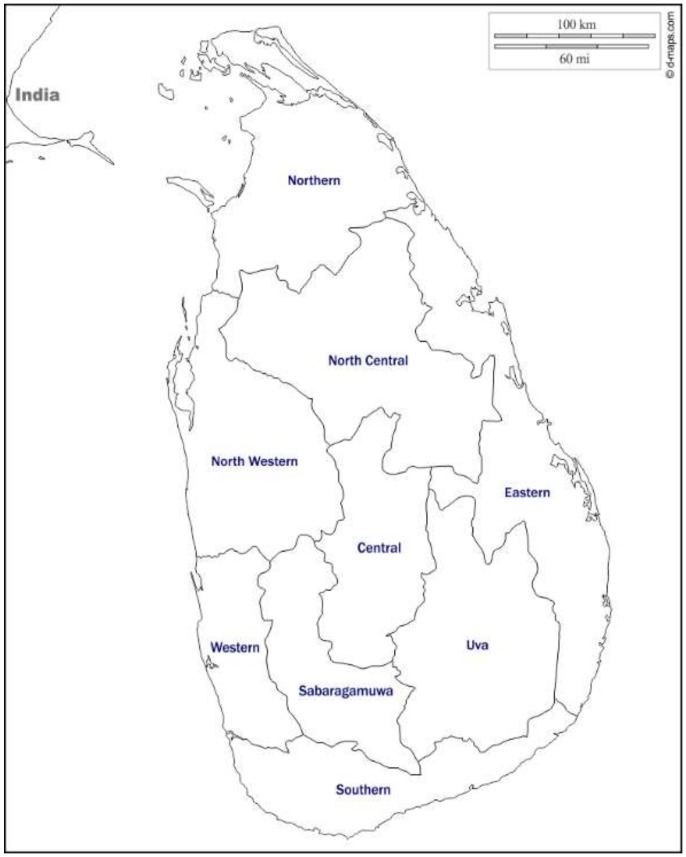
Map of Sri Lanka demarcating nine provinces.

mUIC, TGRs, and HHIS were available for analysis from 4 national iodine surveys (NISs) between 2000 and 2016—NIS2000, NIS2005, NIS2010, and NIS2016 [13,14,15,16]. These NIS used a two-stage stratified cluster-sampling technique as specified by the WHO, UNICEF, and IGN [17,18]. During each NIS, the same team of field investigators visited all nine administrative provinces of the country to detect goitres by palpation, and collected urine from 6–12-year–old schoolchildren, and salt from their households and drinking-water samples from the household or school locality. Figure 1 illustrates the map of Sri Lanka demarcating 9 provinces. All four national studies were carried out to ascertain provincial variation. A total of 16,910 schoolchildren of 6–12 years of age were studied in the four surveys and included in the final analysis (Table 1). Furthermore, we had available data for analysis from the national micronutrient study in pregnant women in 2015 (MNSPM2015) (Table 2) [19].

### 2.2. Indicators of Population Iodine Status

Three primary indicators of population iodine status were considered, and we used the methodology described below to assess the outcomes of the USI programme: (i) mUIC was measured by ammonium persulfate digestion with spectrophotometric detection of the Sandell–Kolthoff reaction in a laboratory certified by the EQUIP programme [20,21,22]; (ii) TGR—the grading of goitres was done by palpation by the same team utilising the classification recommended by the WHO, UNICEF, and IGN [3,18]: (a) “no goitre”—thyroid not palpable or visible; (b) “goitre present”—thyroid palpable not visible or palpable and visible; and (iii) iodine content in salt: titration method to measure the iodine content of salt certified by a regional iodine laboratory [3,18]. Geographical location (province), iodine in drinking water, and household salt were measured to estimate their influence on optimal iodine consumption. Iodine levels in drinking water at the household level and school localities were tested using ammonium persulfate oxidation [20].

## 3. Data Analysis

The following definitions were used for classifying population iodine nutrition status [22]. (i) Median UIC: (a) adequate mUIC—150–299 µg/L (pregnant women) and 100–299 µg/L (schoolchildren); (b) excessive mUIC—≥300 µg/L; and (c) iodine sufficiency—<20% samples should have mUIC of <50 µg/L. (ii) Household salt iodine (HHIS) content: we classified salt iodine content as follows. (a) <5 mg/kg—non-iodised; (b) 5–14.9 mg/kg—inadequately iodised; (c) 15–30 mg/kg—adequately iodised; and (d) >30 mg/kg—over-iodised. (iii) Iodine content in drinking water: iodine in drinking water was classified as follows. (a) <5 mg/kg—no iodine; (b) 5–14.9 mg/kg—low iodine; (c) 15–30 mg/kg—moderate iodine; and (d) >30 mg/kg—high iodine [23,24].

Statistical analysis was performed using SPSS (IBM version 24). Data that were not normally distributed were expressed as median and interquartile range (IQR) unless otherwise stated. The Mann–Whitney U–test was used to compare data between the two groups. The Kruskal–Wallis test (nonparametric analysis of variance (ANOVA)) was used to assess the significance of differences between more than two groups. Categorical variables were analysed using the chi-squared test for trend; a p–value of <0.05 was considered statistically significant.

## 4. Results

(i) mUIC was consistently in the adequate or iodine-sufficient range in all four national iodine surveys of 2000–2016. There has been a significant increase in mUIC, but still within the adequate range in surveys between 2000 (145.3 (84.6–240.4)) and 2016 (232.5 (159.3–315.8)); *p* = 0.000). There has also been a significant reduction in the percentage of schoolchildren with mUIC < 50 µg/L (2.7% in 2000 vs 1.6% in 2016; *p* = 0.000). As shown in Table 2, the mUIC of pregnant women was also in the adequate or iodine-sufficient range (157.7 (228.9–337.6) µg/L) at the national level, and in the second and third trimesters 217.5 (115.6–313.0), and 273.1 (228.9–337.6) µg/L; p < 0.000). Table 3 shows there is regional variability in mUIC levels in children of 6–12 years of age (297.3 *vs*. 178.8 µg/L in 2016; *p* = 0.000). It was significantly higher in the northern and north–central provinces when compared to the rest of the country since 2005.

(ii) There was significant reduction in TGR by palpation between surveys done in 2000 (18.0%) and 2016 (1.9%; *p* = 0.000; Table 1).

(iii) The iodine content of HHIS was only measured since 2005, and since that time, over 95% of all HHIS has contained at least some iodine (>5 mg/kg). The percentage of HHIS with adequate iodine concentrations (defined as 15–30 mg/kg) showed a significant increase—47.7% in NIS2005 *vs*. 63.5% in NIS2016 (*p* = 0.000). Furthermore, only 3.1% had a salt content of <5 mg/kg (non-iodised) in the last survey in 2016. The prevalence of over-iodised salt (>30mg/kg) significantly fell from 43.5% in 2005 to 15.0% in 2016 (*p* = 0.000; Table 1). HHIS was less than 90% at the national level, and in all provinces in 2010 and 2016 except for the central and eastern provinces. In 2016, the interprovincial difference of median iodine content in HHIS was between 18.0 and 27.5 mg/kg (Table 3).

(iv) Median iodine content of drinking water was 33.4 (12.3–66.8) µg/L. Wide variation was observed between provinces (8.3 (4.6–29.0) vs 75.5 (48.4–102.5) µg/L; *p* = 0.000) in the uva and north–central provinces, respectively (Table 4).

**Figure 2:**
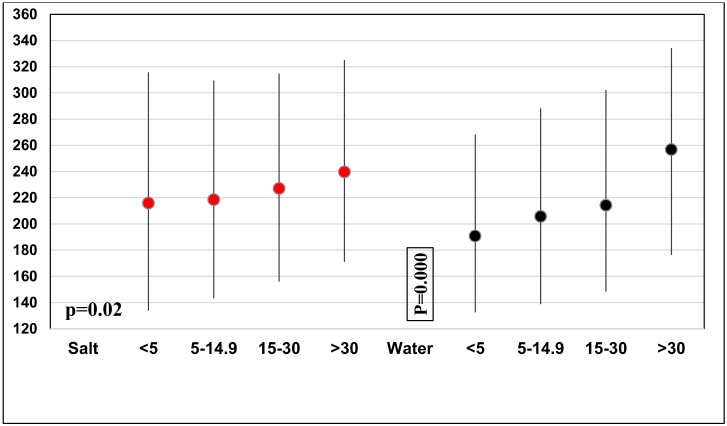
Median urine iodine concentration (IQR) and its relationship with iodine concentrations in household salt and drinking water in school children aged 6–12 years in 2016.

Figure 2 provides a graphical representation of the data on median UIC of children aged 6–12 years in 2016, stratified by the iodine content in HHIS and in drinking water. These data are noteworthy since the mUIC was within the optimal range in all subgroups, including those households of which the iodine content in HHIS was <5 ppm or in the range of 5–14.9 ppm, suggesting that the consumed iodine in HHIS is not the exclusive diet source of iodine. There was a significant increase in median UIC with increasing iodine concentrations in drinking water (*p* = 0.000).

## 5. Discussion

USI was first implemented in Sri Lanka in 1995. We demonstrated in this retrospective review of data from four national iodine surveys of over more than two decades of continuous salt iodisation that (i) mUIC has consistently been in the adequate range with a sequential increase within safe and recommended limits; (ii) the goitre-prevalence rate to palpation in children between 6–12 years significantly decreased between 2000 and 2016 (18.0% to 1.9%; *p* = 0.000); and (iii) the percentage of adequately iodised household salt samples significantly increased during this period (47.7% in 2005 *vs*. 63.3% in 2016; *p* = 0.000), and its household consumption remains satisfactory (Table 1 and Table 3).

These indices of population iodine nutrition favourably reflect the success of the USI programme enforced by successive governments of Sri Lanka, having adequate iodine status at the national level and in most provinces (Table 1 and Table 3). Furthermore, there has been a recurrence of iodine deficiency in several countries where iodine-deficiency disorders (IDDs) were eliminated with USI because of inadequate monitoring of their USI programmes [25,26,27,28,29]. Strict monitoring is essential in sustaining proper iodine nutrition in countries that adopt USI [28].

However, there is a need for caution. (a) The median UIC of pregnant women is only marginally above the recommended cut off of 150 µg/L, and iodine-insufficient in the first trimester (102.3 (61.7–147.1) µg/L (Table 2)). There was a remarkable improvement in the iodine status of pregnant women compared to 2011 (113.7 µg/L) [30]. There was also a significant minority of pregnant women (nearly 10%) who had a median UIC of <50 µg/L. This is an important population group, and inadequate iodine delivery to this group may have important long-term consequences, particularly regarding the intrauterine development of the brain, central nervous system, and physical growth [29]. (b) The median UIC of schoolchildren in the northern and north–central provinces in 2016 approached 300 μg/L. In these two areas at risk of iodine excess, iodine content in drinking water was the highest among those provinces (Table 4). Other countries’ experience with high iodine content in drinking water should be reviewed [31,32]. (c) Some regional variability in mUIC was observed over the course of the programme, but in the most recent survey, the range was between 178.8 and 297.3 µg/L, all within the optimal range. The reasons for regional variability of median UIC have not been investigated in detail, but need to be noted (Table 2). There is a clear need, therefore, to closely monitor these groups in the future, with periodic well-designed and more elaborate studies. However, we acknowledge that these UIC assessments were done on single “spot” samples of urine and may not be truly representative of the iodine-nutrition status of each individual in these communities [28].

We also showed that the supervision and monitoring of salt iodisation has improved over two decades of USI. The percentage of samples delivering adequate levels of salt at the consumer level (i.e., 15–30 mg/kg) increased from 47.5% in NIS2005 to 63.3% in NIS2016 (*p* = 0.000), while at the same time, the percentage of over-iodised salt samples has significantly decreased (*p* = 0.000; Table 1). The percentage of households using adequately iodised salt was less than 90% (the WHO goal for USI) at the national level and in seven out of nine provinces (Table 3). However, it showed that the median HHIS content in provinces was between 18.0 and 27.5 mg/kg, confirming that household iodised salt was providing a significant amount of iodine to the diet [33].

Despite a <90% of households consuming adequately iodised salt, there has been an increase in mUIC, and some provinces in the country consistently showed a high level of mUIC. Daily mean per capita salt intake of Sri Lankans was reported as 8.3 g (CI: 7.9, 8.8) in 2012 [34]. We also need to be aware of the contribution of other sources of iodine contributing to population iodine nutrition, e.g., drinking water, processed foods, or condiments, which are being manufactured with iodised salt, as well as some iodine in foods. Our results indicated a positive association between iodine status in schoolchildren and water iodine concentration, although the major contributor to iodine intake is iodised salt in the diet (Figure 2). In fact, over 95% of households have consistently had access to iodised salt since 2005. A similar contribution was observed in other countries [24,32]. There is a need to adjust the recommended level of HHIS, and to explore the iodine supply through different dietary sources and the geological assessment of soil iodine content for future monitoring.

IGN/UNICEF recommends that the optimal iodine intake, as measured by the median UIC for school-age children, should be <300 µg/L, while the mUIC among pregnant women should be <500 µg/L [22]. Thus, the current salt-iodisation programme is having its desired impact and not placing the Sri Lankan population at risk for iodine excess, as described in the previous study [33]. The salt-iodisation programme needs to be consistently monitored so that the level of iodine in all edible salt, including that used at the household level as well as in processed foods and condiments, leads to an optimal intake. As salt-reduction efforts are implemented, there may be a decline in overall salt consumption, in which case the government may need to accordingly adjust the recommended salt iodine level to ensure that public-health strategies of iodine-deficiency prevention, salt reduction, and reduction in NCDs are realised.

Despite adequate iodine nutrition among schoolchildren, iodine nutrition among pregnant women remains just above the cut-off levels in the country. There is a need to focus on pregnant women for continuous monitoring while sustaining the iodised-salt programme.

This study has several strengths. (a) Data availability from a large number of 6–12-year-old schoolchildren (16,910 in total); (b) uniform methodology for UIC assessments over the period of review in a single laboratory with stringent external quality control; (c) permanent health staff used as a single team in all four studies and goitre palpation; (d) minimising variability in urine- and salt-assay methodology using the same protocols developed by the UNICEF, WHO, and IGN. However, the unavailability of pre-USI data for comparison was an inherent shortcoming of this study.

## 6. Conclusions

The iodine nutrition of the population has remained optimal and stable in Sri Lanka during more than two decades of continuous salt iodisation after its introduction in 1995. However, we recommend the close and careful monitoring of pregnant women and schoolchildren in view of the data we presented. The delivery of salt to consumers has improved and is adequate in the majority. The contribution of dietary sources other than salt needs to be assessed in well-planned studies.

## Figures and Tables

**Table 1:** Median urine iodine concentration (mUIC), goitre prevalence, and household salt iodine consumption in schoolchildren aged 6–12 years in 2000–2016. TGR, total goitre prevalence rate; HHIS, household salt iodine; IQR, interquartile range.

Surveys	UIC (µg/L)		TGR ^3^	HHIS (%) ^4^			
	% < 50 ^1^	Median (IQR) ^2^	%	<5	5–14.9	15–30	>30
NIS–2016 (n = 5000)	1.6	232.5 (159.3–315.8)	1.9	3.1	18.4	63.5	15.0
NIS–2010 (n = 7401)	6.7	163.4 (99.1–245.1)	4.4	4.6	27.1	52.5	16.1
NIS–2005 (n = 1879)	7.4	154.4 (90.3–252.6)	3.8	0.0	8.7	47.7	43.5
NIS–2000 (n = 2628)	2.7	145.3 (84.6–315.8)	18.0	–	–	–	–

Note: ^1–4^ *p* = 0.000. (- No data)

**Table 2:** Median UIC in pregnant women in three trimesters (national micronutrient study in pregnant women in 2015, NNMSPM2015).

Trimesters	UIC (µg/L)		No
Period of Amenorrhea (POA)	% <50 ^1^	Median (IQR) ^2^	
First trimester (≤12 weeks of POA)	17.0	102.3 (61.7–147.1)	447
Second trimester (13–28 weeks of POA)	6.2	217.5 (115.6–313.0)	339
Third trimester (>28 weeks of POA)	0.0	273.1 (228.9–337.6)	176
**Overall**	**10.1**	**157.7 (91.2–256.4)**	**962**

^1,2^*p* = 0.000.

**Table 3:** Regional variations of key indicators of population iodine nutrition in 2000–2016.

Province	Median Iodine Content in Salt (IQR; mg/kg)			Adequately Iodised HHIS (%)			Median UIC (IQR) (µg/dL)			
	2005 ^1^	2010 ^2^	2016 ^3^	2005 ^4^	2010 ^5^	2016 ^6^	2000 ^7^	2005 ^8^	2010 ^9^	2016 ^10^
Western	28.5 (22.3–37.9)	21.2 (13.2–27.5)	19.0 (14.8–25.4)	96.1	70.0	71.6	151.4 (92.8–238.1)	142.2 (96.7–197.7)	168.4 (11.7–231.5)	233.1 (166.7–313.3)
Southern	32.7 (23.2–41.7)	21.2 (11.6–27.5)	21.2 (13.8–25.4)	94.4	66.7	70.2	122.4 (74.2–178.9)	111.0 (69.9–189.5)	123.3 (74.3–203.0)	201.3 (121.5–289.9)
Central	27.5 (20.6–34.9)	22.2 (14.8–27.5)	27.5 (21.2–34.9)	97.4	74.0	91.0	96.2 (61.6–149.1)	144.7 (83.8–211.9)	168.2 (104.1–247.4)	220.7 (168.3–286.4)
Northern	19.0 (14.8–26.9)	14.8 (7.4–23.3)	22.2 (18.0–26.5)	74.3	48.3	83.6	139.5 (74.1–247.4)	283.4 (182.8–403.1)	203.8 (124.6–292.1)	297.3 (230.4–355.4)
Eastern	29.0 (21.6–45.9)	23.3 (16.9–28.6)	23.3 (20.1–26.5)	90.6	78.5	91.2	231.3 (152.9–328.3)	160.4 (94.5–250.9)	173.2 (110.9–241.7)	233.8 (159.5–323.5)
North Western	28.0 (22.7–35.8)	19.0 (9.4–25.4)	19.3 (12.7–24.3)	93.6	60.6	68.1	122.5 (76.6–190.9)	152.8 (98.7–221.3)	151.7 (93.4–228.1)	229.4 (155.9–318.6)
North Central	28.6 (20.4–40.7)	21.2 (12.7–27.5)	18.0 (12.2–24.3)	90.1	67.7	64.1	135.9 (76.9–204.9)	229.9 (135.2–332.0)	237.9 (164.6–328.7)	278.0 (186.3–327.2)
Uva	28.5 (23.8–30.1)	23.3 (13.8–28.6)	21.2 (16.9–25.4)	94.6	72.9	81.5	181.1 (106.0–320.1)	108.5 (68.4–186.4)	129.3 (78.9–198.1)	178.8 (126.5–259.1)
Sabaragamuwa	32.0 (22.7–41.2)	22.2 (12.7–29.6)	22.2 (18.0–27.5)	92.4	70.7	82.0	194.4 (117.6–304.0)	109.0 (69.3–205.8)	121.1 (69.7–187.0)	217.5 (148.7–305.0)
Sri Lanka	28.0 (20.6–38.6)	21.2 (11.6–27.5)	21.2 (15.9–26.5)	91.4	67.6	78.0	145.3 (84.6–240.4)	154.4 (90.3–252.6)	163.5 (99.1–245.1)	232.5 (159.3–315.8)

Note: ^1–10^ *p* = 0.000.

**Table 4:** Regional variations of median iodine content of drinking water in 2016.

Province	No	Median (IQR) µg/L
Western	67	15.6 (4.1–29.1)
Southern	70	19.1 (15.3–29.9)
Central	68	18.0 (5.7–44.6)
Northern	78	53.4 (28.9–79.4)
Eastern	189	33.3 (17.0–69.6)
North Western	122	39.9 (9.4–61.4)
North Central	170	75.5 (48.4–102.5)
Uva	62	8.3 (4.6–50.4)
Sabaragamuwa	108	31.3 (15.1–50.4)
Sri Lanka	934	33.4 (12.3–66.8)

Note: *p* = 0.000.
